# Exploring patient‐reported outcomes following percutaneous coronary intervention: A qualitative study

**DOI:** 10.1111/hex.12636

**Published:** 2017-11-12

**Authors:** Darshini R. Ayton, Anna L. Barker, Geeske M. E. E. Peeters, Danielle E. Berkovic, Jeffrey Lefkovits, Angela Brennan, Sue Evans, John Zalcberg, Christopher Reid, Johannes (Just) Stoelwinder, John McNeil

**Affiliations:** ^1^ Department of Epidemiology and Preventive Medicine School of Public Health and Preventive Medicine Monash University Melbourne Vic Australia; ^2^ Trinity College Dublin Trinity College Institute of Neuroscience Dublin Ireland; ^3^ NHMRC Centre for Research Excellence in Cardiovascular Outcomes Improvement Curtin University Perth WA Australia

**Keywords:** patient perspectives, patient‐centred care, patient‐reported outcome measures, patient‐reported outcomes, percutaneous coronary intervention

## Abstract

**Background:**

Percutaneous coronary intervention (PCI) is a common cardiac procedure used to treat obstructive coronary artery disease. Patient‐centred care is a priority in cardiovascular health having been shown to increase patient satisfaction, engagement with rehabilitation activities and reduce anxiety. Evidence indicates that patient‐centred care is best achieved by routine collection of patient‐reported outcomes (PROs). However, existing patient‐reported outcome measures (PROMs) have limited the patient involvement in their development.

**Aims:**

To identify and explore outcomes, patients perceive as important following PCI.

**Methods:**

A qualitative design was adopted. Eight focus groups and five semi‐structured interviews were conducted with 32 patients who had undergone PCI in the previous 6 months. Outcomes were identified and mapped under the U.S. Food and Drug Administration (FDA) patient‐reported outcome (PROs) domains of feeling (physical and psychological outcomes), function and evaluation. Inductive and deductive analysis methods were used with open, axial and thematic coding.

**Results:**

Consistent with prior studies, patients identified feeling and function outcomes such as reductions in physical and psychological symptoms and the ability to perform usual activities as important. Participants also identified a range of new outcomes, including confidence to return to usual activities and evaluation domains such as adverse effects of medications and the importance of patient communication.

**Conclusion:**

The findings of this research should be considered in the design of a cardiac PROM for PCI patients. A PROM which adequately assesses these outcomes can provide clinicians and hospital staff with a foundation in which to address these concerns or symptoms.

## 1 INTRODUCTION

Percutaneous coronary intervention (PCI) is a common cardiac procedure used to treat obstructive coronary artery disease. It involves the use of balloon catheters and specially designed coronary stents to alleviate coronary narrowing and promote effective blood flow to the heart.^1^ In 2010, approximately 150 PCIs per 100 000 population were performed in Australia.^2^ In the UK in 2013, the rate of PCI was 144 PCI per 100 000 population.^3^ Having insight into patient health and well‐being post‐PCI can inform the tailoring of health services, post‐procedural support resources and follow‐up care—important strategies in patient‐centred care.

Patient‐centred care has been shown to increase patient satisfaction, engagement with rehabilitation activities, and reduce anxiety.^4^, ^5^ Patient‐centred care involves respecting patients’ preferences, providing physical and emotional support, effective communications, tailoring of care and involving family and friends in care decisions.^4^ The U.S. Food and Drug Administration (FDA) states that patient‐centred care should be informed by routine collection of patient‐reported outcomes (PROs). PROs are an assessment of health or well‐being from the perspective of the patient without interpretation by a clinician or health provider^6^ collected through patient‐reported outcome measures (PROMs). To capture the patient perspective, PROMs should be developed by obtaining patient input in the identification of outcomes and items for inclusion on the measure, and the wording of the items.^7^

The FDA developed three domains for PROs^6^:

1. *Feeling*: How the patient feels physically and psychologically after medical intervention;
2. *Function*: The patient's mobility and ability to maintain their regular routine;
3. *Evaluation*: The patient's overall perception of, and what contributed to, the successfulness or failure of their procedure.

In the existing literature examining recovery post‐coronary angioplasty, feeling and function domains were evident in physical outcomes and psychological outcomes. Earlier studies identified pain as a common physical outcome amongst patients^8^, ^9^, ^10^; however, participants in more recent studies prioritize psychological outcomes such as anxiety, emotional shock and uncertainty about the future over pain.^11^, ^12^, ^13^ These differences may reflect changing procedural techniques including drug‐eluting stents and user‐friendly operating tools^14^, as well as social norms. New techniques may reduce pain experienced by patients, and mental well‐being was rarely considered an important element of recovery during the 1990s.^15^

The evaluation domain is demonstrated in prior research highlighting the relationship between procedural expectations and PROs and the importance of doctor‐patient communication in perceived successful recovery.^8^, ^9^, ^10^, ^11^, ^12^, ^13^, ^16^, ^17^, ^18^, ^19^, ^20^ PROs describing elements of cardiac rehabilitation and patient support services that aide recovery, including stress management techniques and modifying health habits, are also evaluated in the literature.^12^, ^18^

To adhere to, and achieve the benefits of patient‐centred care, it is essential that PROMs accurately capture outcomes specific to the patient population.^7^ Only one condition‐specific PROM─the Coronary Revascularisation Outcomes Questionnaire (CROQ)─was developed using methods that captured patient's perspective. Semi‐structured interviews were conducted with ten percutaneous transluminal coronary angioplasty (PTCA) patients asking about their experiences of PCTA. A review of the literature identified 10 articles related to PROs for PCI patients: nine qualitative studies and one mixed methods study. Five studies conducted interviews;^10^, ^12^, ^19^, ^20^, ^21^ four studies conducted focus groups;^8^, ^9^, ^16^, ^18^ and one study consisted of semi‐structured interviews and the completion of pre‐existing PROMs (Illness Perception Questionnaire, Hospital Anxiety and Depression Scale and the General Health Questionnaire‐12).^11^ In some of these studies, the data collection has been deductive^8^, ^9^, ^11^, ^18^ or focused on one aspect of the patient experience such as provision of health information or the process of preparing for the procedure.^10^, ^16^, ^21^ The deductive use of pre‐existing measures or focused topic areas to guide the focus groups and interviews may have restricted the researchers’ ability to ask follow‐up questions and limited the participants’ ability to provide a well‐rounded account of recovery outcomes. One study employed an inductive data analysis technique; however, there were only 11 participants in the study.^12^ Interviews with PTCA patients were conducted approximately 4 weeks post‐discharge. Long‐term concerns arising from the procedure such as anxiety were not explored, and the study does not state at what point of PROM development PTCA patients provided input.

The aim of this research was to identify and explore outcomes patient perceive as important following PCI using inductive approaches to qualitative data collection and analysis methods.

## 2 METHODS

### 2.1 Participants

Patients aged 18 years or older who had undergone a PCI in the previous 6 months were eligible to participate. Patients who were unable to participate in a focus group due to cognitive decline or limited knowledge of the English language were excluded. Recruitment was via four hospitals in Victoria, Australia and a private health insurer. Eligible patients were sent an invitation letter via the hospital or insurer and asked to contact the researchers if they were interested in participating. A total 1154 invitations were sent between March and August 2016 resulting in contact from 90 (8% response rate) interested patients. Thirty‐four patients were ineligible to participate based on the timing of their PCI, and 14 were unable to attend a scheduled focus group or interview based on timing, location or unforeseen circumstances. Eight participants declined to participate and two participants were lost to follow‐up.

### 2.2 Data collection

Eight focus groups and five interviews were conducted with 32 participants. Prior to the focus groups, participants completed questions about demographic characteristics and health status. The US Food and Drug Administration (FDA) categorizes PROs into three domains: feeling (how the patients feel physically and psychologically), function (mobility, ability to perform daily tasks) and evaluation (the patient's overall perception of the successfulness of their procedure and care).^6^ Participants were asked three open questions which related to these domains:

1 From your perspective, what would be considered a successful outcome of the procedure?
  - *Probing questions*: Did the procedure meet your expectations? How do you define whether the procedure was successful?
2 Can you tell me how you felt after the procedure?
  - *Probing questions*: How did you feel 1 week after and how does that compare with how you feel now?
3 After your procedure, can you tell me about your ability to do your daily activities?
  - Prompt for activities including gardening, housework, personal care, work‐related and family‐related tasks.
  - *Probing questions*: Did you attend cardiac rehabilitation? Can you tell us about your experience of cardiac rehabilitation? What impact has medication had on your recovery? Can you describe any lifestyle changes you have made since your procedure?

Data collection was iterative and probing questions were asked depending on the responses and the findings of previous focus groups and interviews. Focus groups and interviews were audio‐recorded, and notes were made during and after the data collection encounters.

### 2.3 Analysis of data

Transcripts were entered into NVIVO prior to coding. Inductive and deductive coding methods were adopted using a process of open, axial and thematic coding. Open codes were generated by looking for initially captivating concepts from PCI patients. Axial coding was conducted to connect common outcomes identified by participants.^22^ Using deductive coding, outcomes that correspond to feeling, function and evaluation were identified. Inductive coding was applied to identify emerging themes from the participants not previously captured in the literature review. Coding and data analysis was conducted by two researchers (DA and DB) to increase the rigour of the analysis. DA and DB compared codes for consistency and discussed discrepancies with AB. Consensus was reached on all codes. The codes and themes were presented to all the authors for feedback.

## 3 RESULTS

Patient characteristics are shown in Table 1. The majority of the participants were male (83%), had private health insurance (77%), lived with a partner/spouse (67%) and were retired (67%). The mean age of the participants is 70 (SD 9.1) years of age. The patient characteristics compared to Victorian Cardiac Outcomes Registry (VCOR) are shown in Table 2. The age of our sample is slightly younger; however, the proportion of females and those with a diagnosis of myocardial infarction (MI) is similar between both groups. Patients with private health insurance are substantially higher in our sample; however, this is due to recruitment occurring through a private health insurance provider.

**Table 1:** Participant demographics

Demographics	N = 32	%
Male	25	78
Age^a^		
50‐59	3	9
60‐69	12	38
70‐79	10	31
80‐89	6	19
Living status		
Partner/spouse	21	66
Partner/spouse and child/children	3	9
One or more other adult relatives	1	3
One or more children aged 18+	1	3
I live alone	6	19
Education level		
No formal qualifications	1	3
School	2	6
High School	6	19
Certificate/Diploma	8	25
University Degree	8	25
Higher University Degree	5	16
Other	2	6
Employment status		
In full‐time paid work	4	13
In part‐time/casual paid work	3	9
Work without pay	1	3
Retired	22	69
Other (volunteer, self‐employed)	2	6

a One participant did not disclose their age.

**Table 2:** Study sample characteristics compared to VCOR

	Study sample	VCOR
N	32	8214
Age (Mean ± SD)	69.8 ± 9.1	65.7 ± 11.9
Females (%)	22	23.1
Private health insurance (%)	75	36.9
Diagnosis of MI (%)	25	22

The main themes that arose from the data have been mapped to the domains of feeling, function and evaluation. The themes are presented in Table 3 and described below.

**Table 3:** Important outcomes post‐PCI mapped to feeling, function and evaluation domains

Feeling—Physical outcomes
Reduced breathlessness upon exertion or when performing activities of daily living
Reduction in, or elimination of, pain and discomfort (angina)
Improved energy and reduced fatigue
Feeling—Psychological outcomes
Confidence to perform activities of daily living such as housework, work, …
Feeling anxious
Questioning mortality
Overcoming the cardiac blues (depression)
Function
Ability to complete activities of daily living
Evaluation
Importance of cardiac rehabilitation
Impact of medication on bruising and sleep
Patient communication in managing expectations of intervention and recovery

### 3.1 Feeling: physical outcomes

The feeling domain incorporates the physical and psychological outcomes patients perceive as important after their PCI. Participants prioritized improved physical outcomes as an important measure of recovery. The majority of participants identified three main physical outcomes: reduced breathlessness upon exertion or when performing activities of daily living, reduction in or elimination of pain or discomfort (angina), and improved energy and reduced fatigue. In response to the first open question—how participants knew their procedure was successful—participants identified reduced breathlessness as a key indicator: *“it made a difference in the shortness of breath that I used to have,”* and *“the shortness of breath has gone so it's alright now.”*

While the study did not specifically aim to identify differences in preferences across men and women, it emerged that men tended to describe breathlessness in the context of an activity whereas women described breathlessness as a symptom in isolation.

I know it made a bit of difference in the shortness of breath…I can now walk up a hill without actually having to pant. Participant 0702, M, 55

I'd just stop dead in my tracks because I'd breathe in and I couldn't breathe out Participant 1301, F, 84

Participants used various terms to describe their angina: discomfort, tightness, *“really bad chest pains,” “chest pressure”* and *“continuous pain underneath the sternum.”* Some participants described their angina more explicitly, highlighting the severity of the pain:

I felt like a hammer hit me in the chest. I've never experienced that before. Participant 1942, M, 71

Participants who presented with a “*toothache and inflammation in [the] gum”* were *“a little bit sceptical”* when they were told by health professionals that they had angina. They were unaware that angina presented as pain in multiple parts of the body. Despite not recognizing it as angina at the time, participants described their experience of angina as a feeling of heartburn, reflux or indigestion.

Recognising the pain is an important point because in my head I had reflux, like I know about left‐sided pain and the signs and symptoms of a heart attack, but that's not what I had. Participant 2242, F, 64

Participants stated that they were *“very exhausted”* and *“very tired”* before their procedure; however, this improved post‐procedure:

I used to be quite tired for about six months prior to this and now I've worked out why I was tired. And since then, since I've had the procedure I haven't been tired at all. Participant 0701, M, 71

The majority of participants who identified fatigue as an outcome were aged between 75 and 84 and concluded that experiencing these symptoms was unavoidable due to their age:

With me now it's a symptom pretty much all the time but I guess these guys would be the same way, we're older too. Participant 1943, M, 75

### 3.2 Feeling: psychological outcomes

Psychological outcomes identified by participants included confidence to perform activities of daily living, questioning their mortality and the cardiac blues. The confidence to perform activities of daily living was seen as an important aspect of recovery:

I have to cook and clean, I did all of that and that didn't stop so that was okay and I could manage that workload… once your heart is weak it's weak. But I had the confidence I could do things. Participant 2801, M, 62

I didn't want to drive so you know I got people to drive for me. That was for a couple of weeks. But then afterwards I started doing it all myself. It was confidence. Participant 2306, M, 67

Participants talked about being overly cautious and *anxious* that they weren't capable of physically performing their normal tasks stating that *“you feel like you've been saved, not cured”* and that *“the heart damage still remains there.”*

I was still anxious but not as anxious as the first time. The first time was total cotton wool. I wouldn't even get out of bed Participant 1621, F, 62

Mentally I was still going through a few issues … I was pussyfooting around… I had myself in cotton wool for a while Participant 0201, M, 69

Participants clarified that over time anxiety levels related to the procedure decreased:

I was scared because I'd read something about sometimes stents move … so there were periods when I was anxious about it but then as the weeks have gone on I've become less and less anxious and more confident that everything's fine for now. Participant 2243, F, 58

A few participants questioned their mortality, particularly the younger participants for whom their cardiac event came as a shock:

I was just in a daze for the first week or two. And then maybe you think too much around mortality… start thinking of your family, and what'll they do without me? Participant 0201, M, 69

Certainly you start to question your mortality… I think that's a big one for everybody Participant 0203, M, 62

Female interview participants provided more information relating to depression, reporting that *“it did take me quite a while to recover, I did get quite depressed afterwards”* and that *“I still feel quite low sometimes.”* One participant identified a term employed by the cardiac rehabilitation staff: cardiac blues. As the focus groups were iterative in nature, subsequent focus groups and interview participants were asked if they were aware of the cardiac blues:

Oh definitely. They mentioned it umpteen times that you will feel depressed, there's nothing you can do about it. All of you have gone through a major procedure and you don't know how you'll come out the other end Participant 2306, M, 67

Participants were generally hesitant to discuss their experiences with depression, however, recognized that these symptoms were relevant to *other* patients:

Not necessarily for me but for the group in case anybody was feeling that way Participant 1601, M, 84

In fact there was a guy who felt that way and they organised a separate meeting for him because he needed extra help. You've got a group there but sometimes you get one or two who need extra help. Participant 1604, M, 61

Older participants indicated that they were dealing with other health conditions that may have complicated their recovery after the PCI and impacted on their psychological well‐being.

You're getting older, you get things like arthritis and you're having injections here and there and everywhere and all your teeth start to ache, wearing hearing aids, I'm wearing glasses and all these things don't help to make you bright and cheerful Participant 1301, F, 84

### 3.3 Function

The function domain encompasses the patient's mobility and ability to maintain their regular routine. Participants were concerned that their procedure would render them *“unable to do what you have to do.”* As a result, participants described their capabilities to do and participate in activities of daily living as a key outcome of their procedure:

“I'm carrying on, I do everything. I walk around, the whole works. I do all the domestics… I'm doing exactly what I was doing before.” Participant 2245, M, 76

One male participant highlighted concerns of not being able to fulfil his family obligations after the procedure. While these were described as daily chores—they were the chores and daily activities that the participant felt responsible for in the family unit and hence worried about not being able to perform.

“I guess you feel sometimes that you won't be able to do what you imagine you have to do. You can't fulfil your obligations in other words. That does concern me.” Participant 1943, M, 75

This was also reflected in the sentiments of a few women who found it difficult to refrain from doing housework.

“[Husband] took the few days off… he wouldn't let me do anything so I think after a week I was getting a bit agitated with stuff. So I started to load the dishwasher and unload the dishwasher” Participant 1621, F, 62

Many participants viewed their ability to adhere to their usual routine as the “*ultimate*” post‐procedure outcome. Nonetheless, participants were realistic about the time required to achieve this goal:

“You'll think you can do things and you'll find out you can't but you will have set yourself back a couple of weeks trying.” Participant 2802, M, 67

Participants did raise concerns about physical, laborious household activities, such as *“vacuuming the house,”* “*wiping kitchen benches,*” *“bleaching the toilets”* and *“putting out the rubbish bins.”*

A few of the younger men described reducing their work commitments or taking longer to get back to their usual work activities than they anticipated.

“I actually cut back my workload by probably three quarters.” Participant 1803, M, 58

Leisure activities were also put on hold while participants recovered from their procedure. Therefore, an important outcome of recovery was being able to socialize and do activities they enjoy.

“It took about three weeks, I guess, once I got out of hospital for me to be able to comfortably walk, ride a bike… I love all of that stuff.” Participant 2801, M, 62

“I do that as much as I can but certainly I don't get out much” Participant 1301, F, 84

### 3.4 Evaluation

The evaluation domain captures the patient's overall perception of aspects that contributed to the successfulness or failure of their procedure, including post‐procedural care strategies. The main themes for the evaluation domain included attendance at cardiac rehabilitation, the side‐effects of medication prescribed post‐procedure and patient communication. The majority of participants reported attending cardiac rehabilitation and felt that it was beneficial. Participants stated that they had been *“fortunate enough to go to rehab”* and that it *“helped tremendously.”*

Participants also thought that attending cardiac rehabilitation provided them with the opportunity to understand their experiences through connecting with others who had gone through the same procedure:

It was just interesting to talk to other people who had the same problems. Participant 0203, M, 62

The information provided in cardiac rehabilitation led to life style and behavioural changes for a number of participants.

The two things that stood out for me were making sure that you get exercise every day and have a look at your diet. I've changed my diet, I'm eating more fresh fruit, I'm not eating as many of my wife's homemade biscuits as I used to. And exercise, I've joined a group where they set exercises for me personally because I've got a bit of a back problem. Participant 2246, M, 86

Participants identified side‐effects to the medication which impacted on their recovery and well‐being, particularly bruising, dizziness and disturbed sleep.

“Sleep is difficult… because of the medication” Participant 1944, M, 65

The disturbed sleep led to feelings of tiredness and fatigue. Dizziness was also attributed to the medication.

“I do get dizzy but I believe it's the blood thinners that are doing it” Participant 0201, M, 69

Participants stated that they were also surprised at the severity of bruising caused by prescribed medication:

I've never been on a blood thinner before and so I was a bit shocked at some of my bruising because I thought bloody hell, can I bleed to death? Participant 2243, F, 58

Participants felt that many of the issues described above in relation to the side‐effects from medication could have been mediated with improved patient communication. This would have helped understand symptoms, expectations of recovery, impact of the procedure and corresponding management.

If somebody had said because you're on blood thinners and you're also on this drug now that's new and anti‐clotting and you're probably going to be a little bit more black and blue than normal. Because I got a hell of a fright when I'd seen the left leg. Participant 1621, F, 62

Doctors played an important role in explaining angina to their patients and patients appreciated talking to doctors about their concerns or symptoms.

Whenever I feel a bit of pain… I go straight to my cardiologist who doesn't argue with me… And I've never been wrong, he accepts what I say, he is very, very good. Participant 1942, M, 71

Female participants in particular emphasized the importance of communication with their cardiologist in relation to recognizing cardiac symptoms, due to the *“difference between the different sorts of signs and symptoms that people have and often women don't have.”*

## 4 DISCUSSION

In this study, the outcomes patients report as important following PCI were explored. The qualitative design allowed for the in‐depth study of patient experiences articulated in their own words—an important aspect in the identification and development of PROs.^6^ A range of outcomes were identified and mapped under the FDA PROs domains of feeling (physical and psychological outcomes), function and evaluation. Physical outcomes included reduced breathlessness when exerting oneself or undertaking activities, reduced or no angina, reduced fatigue and improved energy. Psychological outcomes identified were anxiety, questioning mortality and cardiac blues (depression). A unique theme identified in this study was the concept of confidence to continue with usual activities. Participants described being cautious, anxious and uncertain in their abilities after the PCI; however, their confidence increased over time. The ability to complete activities of daily living (ADLs) was the main function outcome. Evaluation outcomes related to the benefits of cardiac rehabilitation, the adverse effects of medication and the importance of patient communication.

The findings correspond and extend on the results of previous studies.^8^, ^9^, ^11^, ^20^ Angina is a typical outcome reported in prior studies.^10^, ^12^ However, this study highlighted the need to understand the different ways angina is experienced. Many participants described angina as discomfort instead of pain and females in particular described the sensation as reflux or heartburn. In this study, some participants did not describe depression symptoms yet highlighted that this was an issue for other patients. This sentiment was also evident in another Australian study which found that patients acknowledged that other patients may need psychological support after their procedure, yet felt they did not feel they personally require this support.^12^ In this study, mental health and psychological outcomes were considered in more detail by participants in interviews than focus groups. This may be due to the privacy and perceived comfort of an individual interview, compared to the open dialogue generated in the focus groups. The generation of participants in this study are generally less mental health literate and therefore unlikely to state feelings of depression^14^.

Emerging findings from the inductive analysis revealed differences between the genders and age groups in relation to patient experiences post‐PCI. Male and female participants identified similar outcomes but described these outcomes differently. Males primarily described their post‐procedure outcomes within the context of performing daily activities or participating in physical activity, whereas females presented their symptoms in isolation. Age was also discovered as an emerging factor that explained differences in the narrative of symptoms. Older participants viewed many of their symptoms as an inevitable part of the ageing process, whereas younger participants viewed the same symptoms as an adverse effect of medication or comorbid conditions. These findings are important as they can be used to enhance patient‐centred care and the tailoring of health education and management strategies. Further examination of these trends is warranted in a larger quantitative study to test whether these patterns persist in a more representative sample.

Good communication between the health‐care team and patients and the importance of providing information through avenues such as cardiac rehabilitation was emphasized by participants in this study. This ability to engage with a member of the care team face to face has also been highlighted in other studies.^21^ Face‐to‐face communication enables the ability to ask questions, discuss the side‐effects of medications and to manage expectations of recovery. Patient communication is a fundamental element of patient‐centred care.^23^ Throughout the literature, patients have highlighted their concerns about ineffective communication, and how it negatively impacts PROs.^16^, ^21^

This study has engaged with patients early in the development of a new PROM for PCI patients. The inductive method of data collection led to the identification of new patient‐reported outcomes including confidence to perform activities of daily living and the need to highlight discomfort as well as pain as a symptom of angina.

## 5 LIMITATIONS

This study has a number of limitations. The analysis and data collection of this study did not focus on the preceding cardiac condition. While the preceding cardiac condition does not change the type of symptoms the patient experiences, it may influence the extent and severity of reported outcomes. For example, a patient presenting with mild angina will recover in less time than a patient presenting with a myocardial infarction. Deeper exploration of elective versus emergency and private versus public patient PCI outcomes is therefore warranted. Given the unique experiences of individuals, further research on the patient experience of PCI should also extend to culturally and linguistically diverse populations.

The response rate from the invitation letters was just under 10%. This is a similar rate to other studies where cold mailing approaches have been taken to recruit participants. However, it does raise questions of whether the sample was more biased to healthier patients who felt well enough to participate.^24^ The majority of participants in this study were male (78%), and therefore, the sample does not equally represent both genders. However, the sample does correspond to the proportion of male and female patients undergoing PCI in Victoria.^25^, ^26^

Despite these limitations, this study provides important new information regarding patient‐reported outcomes following PCI. The qualitative study design facilitated the patient‐centred nature of the research as it allowed for a detailed, insightful exploration of PROs from the perspective of PCI patients. This is also the first study to adopt the FDA's framework of PROs for cardiac research. Open questioning and inductive methods of data analysis were employed to maximize genuine patient input. Two researchers coded the focus group and interview transcripts to ensure accuracy and increase the rigour of the research. The themes identified in this study have been developed into 10 PROM items that were tested in a sample of 138 patients via a discrete choice experiment survey. This enabled patients to trade off preferences between the outcomes with the aim being to refine the number of outcomes for the PROM. Following the discrete choice experiment analysis, the 10 PROM items were reduced to eight and were piloted in 200 patients in the VCOR registry. A Rasch analysis was conducted to validate the measurement properties of the PROM. The final PROM is a five‐item PROM. The results of these studies will be reported elsewhere.

## 6 CONCLUSION

Being able to perform usual activities without breathlessness, the reduction or elimination of angina pain/discomfort and improved energy and reduced fatigue were important physical outcomes for patients post‐PCI. Lacking confidence in being able to undertake activities of daily living, experiencing anxiety and cardiac blues and questioning mortality were the main psychological outcomes identified. These feeling outcomes align with and extend on previous literature. A PROM which adequately assesses these outcomes can provide clinicians and hospital staff with a foundation in which to address these concerns or symptoms. An important evaluation outcome valued by participants was face‐to‐face communication in a format such as cardiac rehabilitation. Patient information is important to ease anxiety over side‐effects of medication and to manage expectations of recovery.

Overall, patients reported a wide range of outcomes pertaining to feeling, function and evaluation post‐PCI. These should be considered as an important component of tailoring patient‐centred care and in the development of future PROMs.

## ETHICAL APPROVAL

Ethics approval was obtained by the participating hospitals and Monash University.
